# There Is No Quick Fix: Compliance Officers’ Views on Organizational Behavioral Change

**DOI:** 10.1007/s10551-025-06183-7

**Published:** 2025-10-22

**Authors:** Malouke Esra Kuiper

**Affiliations:** 1 Erasmus School of Law, Erasmus University Rotterdam, Burgemeester Oudlaan 50, 3062 PA Rotterdam, The Netherlands; 2 Amsterdam Law School, University of Amsterdam, Amsterdam, The Netherlands

**Keywords:** Compliance officers, Experiential knowledge, Behavioral change, Role modeling, Leadership, Sanctions

## Abstract

Major scandals show the damage that organizational misconduct can do to society. Compliance officers play a crucial role in mitigating organizational misconduct. There is extensive empirical research on how such misconduct can effectively be prevented. However, little is known about compliance officers’ experiential knowledge and how this aligns with these scientific findings. Based on semi-structured interviews with compliance officers, this paper analyzes their experiential knowledge and its alignment with empirical research on a conceptual level and, moreover, on a substantive level by zooming in on two concepts: first, role modeling and leadership, and second, sanctions. It shows that none of the interviewed compliance officers believed in a quick fix for compliance: all identified a combination of concepts at play in mitigating misconduct. The alignment on a conceptual level was broad as there was much overlap between the concepts distinguished by compliance officers and concepts studied in empirical research. The alignment on a substantive level was more superficial, however. Compliance officers only minimally elaborated on how they thought the concepts they identified may affect behavior. This has received more attention in empirical research. These insights provide a first understanding of what compliance officers believe are proper methods to mitigate organizational misconduct and how their ideas align with social scientific findings.

## Introduction

Major scandals such as Purdue Pharma’s drug OxyContin and its contribution to the opioid crisis (Keefe, 2021) or the Volkswagen emission scandal (Jung & Sharon, 2019) show the damage that organizational misconduct can do to society. Nowadays, most companies have so-called compliance programs to prevent organizational misconduct (Soltes, 2021). Compliance management is a relatively new field that has rapidly developed over the last few decades. Over the last 30 years, the focus has changed from compliance as merely liability management toward effectively changing behavior and preventing organizational misconduct (Chen & Soltes, 2018; Soltes, 2021). With this change, the key question has become how compliance management can be effective in helping to mitigate and prevent organizational misconduct.

Scholars from different disciplines, such as white collar criminology, business ethics, organizational psychology, and corporate law, have sought to understand to what extent compliance management can be effective in preventing organizational misconduct (e.g., Andreoli & Lefkowitz, 2009; Kish-Gephart et al., 2010). For example, scholars have assessed to what extent compliance management systems (CMSs) are effective in preventing and reducing organizational misconduct (Coglianese & Nash, 2021; Parker & Nielsen, 2009b; Van Rooij & Fine, 2019). Scholars have also focused on the effectiveness of specific elements of such CMSs, such as codes of conduct (Babri et al., 2021; Kaptein, 2021; Stevens, 2008) and ethics and compliance training (Hess, 2021). To study the effectiveness of compliance management, we can also draw on broader research about why people break or comply with rules generally. This body of work shows that compliance originates from a combination of general factors (Kuiper et al., 2023; Weaver, 2014), including incentives (Shover & Hochstetler, 2005; Simpson & Rorie, 2011; Simpson et al., 2014), social norms and social context (Cialdini & Goldstein, 2004; Vaughan, 1997), and opportunities for misconduct (Benson et al., 2009; Van Rooij & Fine, 2021).

If compliance management practice is organized in accordance with insights from such empirical science, it could be more effective in mitigating and preventing misconduct and avoid the harmful behavior that leads to major scandals. For this to happen, the science must somehow come to guide compliance management practices.

Compliance officers play a crucial role here. They are tasked with mitigating misconduct within organizations and setting up and operating compliance management systems (Treviño et al., 2014). In other words, they are often key players in how organizations manage ethics (Den Nieuwenboer et al., 2025). Most compliance officers received legal training, however, and are only rarely trained in the relevant social science about organizational compliance (Soltes, 2021; Weber & Fortun, 2005). Yet they do develop experiential knowledge through their everyday experience in performing their tasks (Borkman, 1976), and thereby formulate their own intuitive ideas about effective ways to mitigate or prevent misconduct. An important risk, however, is that by relying on their experiential knowledge, these compliance officers may utilize practices that science has proven to be ineffective. This would constitute an important threat to compliance management and its effectiveness at mitigating and preventing organizational misconduct.

So far, we do not know much about the experiential knowledge of compliance officers regarding how they can mitigate misconduct in their organization, or how this aligns with relevant existing scientific knowledge. There is a dearth of research on ethical organizational roles such as compliance officers (Den Nieuwenboer et al., 2025). Nonetheless, studying what compliance officers think and how they work can be insightful for the field of business ethics. Compliance officers help to prevent intentional and unintentional harm caused to a business’ stakeholders. As multiple scandals show, breaches in compliance can result in harm done to business’ stakeholders. For example, the Volkswagen “Dieselgate” scandal shows how a compliance lapse can cause environmental and customer harm (Jung & Sharon, 2019). In 2015, investigators discovered that Volkswagen had installed devices in diesel vehicles to lower emissions during testing and thus cheating emission tests, violating environmental laws in multiple countries. The excess of nitrogen oxide emissions contributed heavily to air pollution, as the emissions of these particular vehicles were 10–40 times higher than allowed (Barrett et al., 2015). Beside environmental harm, customers were also harmed as they were misled about the environmental performance of Volkswagen and resale values dropped. As a result, the company’s share price fell by 30–40% in two weeks (Jung & Sharon, 2019). A second example is that of Purdue Pharma and the opioid crisis. Purdue Pharma was the manufacturer of the prescription painkiller OxyContin. Driven by the desire for profit maximization, the company falsely and illegally marketed their drug as safe to use and at low risk for addiction in comparison to other available medication on the market (Meier, 2003). Today, experts believe that their relentless promotion of this drug contributed to the opioid crisis, which has claimed the lives of around 645.000 Americans as of 2021 (Keefe, 2021). Purdue Pharma sought refuge in bankruptcy in 2019, causing employees to lose their jobs (Crofts & van Rijswijk, 2024). As a result, this compliance breach has harmed patients, communities, and employees.

Compliance officers are tasked to prevent such compliance lapses and therefore the intentional and unintentional harm done. Therefore, studying what compliance officers think and how they work can be insightful for the field of business ethics. Following Richard Rorty’s philosophical pragmatism, we gain a better understanding of ethics by studying the actual human processes where values are formed and challenged: ethics is constructed in practice (Rorty, 1979, 1991, 2006). Den Nieuwenboer et al. (2025) illustrate these processes in their study on ethics and compliance officers (ECOs). They show that ECOs have to engage in the navigation of tension inherent to the role and shifting between different approaches as needed. Their position is shaped by a dual threat dynamic: they are often perceived by others as threatening when they enforce ethical expectations, while they themselves feel their own moral identity under scrutiny. To navigate this, they develop tactics to distribute responsibility and reduce defensiveness, and they frame their roles in ways that allow for ethical growth. In pragmatist terms, these practices are moments of redescription: by reframing what ethics means in a given organizational context, ECO’s make moral claims persuasive and actionable for others. They shape the ethical life of an organization by translating legal norms into decisions and managing (moral) risks (Soltes, 2021). Thus, following Rorty’s philosophy about ethics, their role provides an insightful empirical lens on how organizational ethics are actively constructed, contributing to business ethics scholarship by showing ethics as a contingent, relational practice (cf. Pouryousefi & Freeman, 2021; Visser, 2019).

The purpose of the present study is to gain a first understanding of the experiential knowledge of compliance officers, and to what extent there is an alignment between their experiential knowledge with scientific knowledge on corporate compliance.^1^ Answering these questions will increase our understanding of compliance officers and how they think about mitigating organizational misconduct, as research on compliance officers is sparse (Den Nieuwenboer et al., 2025).

In the next section, a short overview of the relevant literature will be provided. Next, the method used to answer the research questions (i.e., semi-structured interviews) will be explained, followed by the analysis of the interviews. Last, the results will be discussed in view of a broader understanding of the literature.

### Organizational Compliance and Behavioral Mechanisms

Most research on organizational compliance focuses on whether compliance programs and specific elements of such programs are effective in preventing or reducing organizational misconduct. Several studies have looked at compliance programs, also known as compliance management systems (CMSs), as a whole. CMSs consist of multiple elements, often the establishment of professional staff combined with a set of formal internal procedures and management to promote compliance, such as (but not limited to) trainings in policies and procedures, a whistleblower protection program, and procedures for corrective action of noncompliance (Coglianese & Nash, 2021). Despite the popularity of such programs, findings on their effectiveness have been mixed. On the one hand, McKendall et al. (2002) found no evidence in their study of 108 large corporations that compliance programs would lessen legal violations. On the other hand, Coglianese and Nash (2021) concluded in their review that CMSs can be linked to fewer compliance violations and to improvements in risk management, but that the effects are limited. In their study of 999 Australian firms, Parker and Nielsen (2009b) found that most of the CMS elements were ineffective in improving organizational compliance. They concluded that good values and good management are just as important as compliance systems for improving compliance.

Other empirical research has focused on specific elements of compliance programs, such as corporate codes of conduct. Such a code is:

[A] distinct and formal document containing a set of prescriptions developed by and for a company to guide present and future behavior on multiple issues of its managers and employees toward one another, the company, external stakeholders and/or society in general. (Kaptein & Schwartz, 2008, p. 113)

Concretely, this means that the company formulates a set of guidelines to which its employees must adhere, with the aim of ensuring that they comply with relevant rules and regulations. Similar to studies about CMS, studies on the effect of codes of conduct present a mixed picture. One review found that codes can be effective in shaping compliance, but that the success of the code is dependent on the culture and on effective communication (Stevens, 2008). Other reviews and one meta-analysis were less conclusive as they found mixed results (Babri et al., 2021; Kaptein, 2021; Kaptein & Schwartz, 2008).

Another topic on which empirical research has focused is whether ethics and compliance training, for example used to increase adherence to the code of conduct, enhances compliance in organizations (Hess, 2021). From a social psychology perspective, the idea is that training will model appropriate behavior and thereby will promote that modeled behavior (Warren et al., 2014). Thus, the goal of the training is often to teach the employee the standards expected in the organization. It is complex to determine whether trainings are effective because different outcomes may be used to evaluate this: how participants react to the program, what they learn, how the program changes their behavior, or how it changes the organization (Steele et al., 2016). In general, research suggests that face-to-face training is more effective than online programs (Medeiros et al., 2017). Furthermore, it seems that the training is more effective when participants can actively engage and receive case-based instructions rather than just following lectures (Harkrider et al., 2012; Medeiros et al., 2017; Mulhearn et al., 2017; Waples et al., 2009; Watts et al., 2017). Nonetheless, a recent study showed that the effects of training fade after 2 years (Warren et al., 2014).

Apart from the research that is specifically focused on corporate compliance practices, there is also a broader body of empirical research on why people generally obey or break rules. This work provides valuable insights into organizational compliance, as it shows us what mechanisms underlie existing corporate compliance interventions. Different social scientific disciplines have developed theories and theoretical families that explain the behavioral mechanisms through which law potentially affects behavior (Kuiper et al., 2023; Van Rooij, 2021). For example, work on social norms shows how human behavior is deeply embedded in a social context: the more the individual’s social context complies with the rules, the more likely it is that the individual will also comply (Cialdini & Trost, 1998; Cialdini et al., 2006; Goldstein et al., 2008; Schultz et al., 2007). Work on punishment provides insights into the positive and negative effects that punishment can have on future offending behavior (e.g., Kirk & Wakefield, 2018; Loeffler & Nagin, 2022; van Rooij et al., 2025; Walters & Bolger, 2019). And work on legal knowledge concludes that people’s knowledge of rules is often lacking or inaccurate, reducing their compliance (Van Rooij, 2021).

In order for compliance systems to function properly, it is important to have an understanding of the mechanisms that are at play and the interventions through which human behavior can be influenced. More specifically, it is important that the practitioners who work within these systems, the compliance officers, are aware of the different mechanisms that play a role and understand how these mechanisms work. However, in the existing literature, little is known about their understanding of the working of compliance systems and their underlying mechanisms.

### Compliance Officers and Experiential Knowledge

To understand what compliance officers think about these questions, it is first important to consider what their role constitutes. Compliance officers are responsible for ensuring that individuals in an organization, both leaders and employees, abide by the rules in force for their organization (Lenglet, 2012; Treviño et al., 2014). It is their task to ensure that both legal and organizational rules are followed and that the employees and leaders act with integrity (Snell, 2015). In practice, the title and exact responsibilities vary within organizations (Treviño et al., 2014; Weber & Fortun, 2005).

Compliance practice is slowly evolving from a purely legal focus on reducing corporate liability for rule breaking toward a greater focus on steering human behavior to meet the demands of policies, regulations, and society to successfully prevent organizational misconduct (Soltes, 2021). However, most compliance officers are still attorneys by training (Soltes, 2021; Weber & Fortun, 2005). This means that they are not trained in the social sciences that provide the necessary understanding, explanation, and prediction of organizational compliance. Instead, most compliance officers rely on their legal training, complemented by their own experiences, to prevent misconduct within their organization. These experiences^2^ form the basis of their so-called “experiential knowledge” (Borkman, 1976, p. 446).

Experiential knowledge can be defined as “[…] truth learned from personal experience with a phenomenon rather than truth acquired by discursive reasoning, observation, or reflection on information provided by others” (Borkman, 1976, p. 446). There are two important elements of experiential knowledge. The first focuses on the type of information: it entails information gained from personal participation in a phenomenon and based on the individual’s actual experience. This experience is more or less representative of the experience of others with the same problem. The second important element is that the experiences indeed become knowledge. As Borkman (1976) stated, it “denotes a high degree of conviction that the insights learned from direct participation in a situation are truth, because the individual has faith in the validity and authority of the knowledge obtained by being part of a phenomenon” (p. 447).

So far, we lack a proper understanding of the experiential knowledge of compliance officers about the effectiveness of compliance management practices and the underlying mechanisms that shape this. Empirical research studying compliance officers is rather sparse and focuses primarily on the state of practice (Den Nieuwenboer et al., 2025; Krambia‐Kapardis et al., 2019; Treviño et al., 2014; Weber & Fortun, 2005). These studies show that compliance officers face legitimacy obstacles and challenges inside their organization, which complicates their work and the ability to reach their goals. This body of work does not tell us how compliance officers think about mitigating organizational misconduct and which methods they see as appropriate to do so. As such, we do not know whether compliance officers’ own thinking about how they can be effective in their job actually aligns with key scientific insights about this question.

### The Present Study

The aim of the present paper is to research the alignment between compliance officers’ experiential knowledge and scientific knowledge on preventing misconduct. It will focus on three core research questions. First, it will focus on the views of compliance officers on preventing misconduct: how do they think they can do so? In other words, what is the experiential knowledge of compliance officers on changing human behavior? Second, to what extent is this experiential knowledge aligned with social scientific insights on compliance on a general level (i.e., on a conceptual level)? Last, to what extent is the experiential knowledge aligned with scientific knowledge on a specific level (i.e., on a substantive, content level)?

To answer these research questions, a qualitative approach is perceived as most suitable. To assess this, this paper analyzes data from in-depth semi-structured interviews with 21 Dutch compliance officers. This data will allow for an in-depth analysis of the provided answers, and to analyze whether any qualitative patterns appear. Consequently, any statements that may seem to suggest causal claims or statistical patterns, should be read in a qualitative context as such claims cannot be formally tested.

## Methods

Ethical approval for this study was obtained from the Ethics Review Board of the Amsterdam Law School, University of Amsterdam, on March 26, 2020. All participants provided consent before the start of the interview and were asked consent for both the recording and the use of quotes. Participation was voluntary.

### Interview Sample

The sample consists of 21 compliance officers located in the Netherlands. Thirteen participants worked in the private sector and eight worked in the public sector, at 18 different organizations. There was some variety in educational background: 13 participants had studied law, five had a social scientific or economic background, and three had a background other than law or social science. Table 1 provides an overview of interviewees and general information. More information about interviewees cannot be provided in order to protect their identity.

**Table 1:** Interviewees’ details

Participant code	Sex	Type of organization	Education
CO001	Male	Private	Law
CO002	Male	Private	Law
CO004	Male	Private	Other
CO005	Male	Private	Law
CO006	Female	Private	Criminology
CO007	Female	Private	Other
CO008	Female	Private	Law
CO009	Female	Private	Criminology
CO010	Female	Private	Criminology
CO011	Male	Private	Economics
CO012	Female	Public	Other
CO013	Female	Private	Law
CO014	Female	Private	Law
CO015	Male	Private	Law
CO016	Female	Public	Law
CO017	Female	Public	Law
CO018	Male	Public	Law
CO019	Male	Public	Law
CO020	Male	Public	Economics
CO021	Female	Public	Law
CO022	Female	Public	Law

The interviewees were recruited through several techniques. Some of the participants were contacted through the network of the research team via email. After these initial contacts and participants, we used snowballing (i.e., assessing participants through contact information provided by other participants, Noy, 2008). This technique is considered to be the most appropriate technique for reaching this group, because compliance officers can be difficult to approach for research in light of their role and responsibilities, meaning that these interviews can be seen as elite interviews (Dexter, 1970; Harvey, 2011). To limit the risk of distortion, several separate chains were initiated (Atkinson & Flint, 2001).

### Data Collection

Data were gathered through semi-structured interviews. Prior to the interviews, a question guide was created with the research team. The development of the question guide consisted of multiple phases (e.g., Kallio et al., 2016). During the first phase, a preliminary semi-structured interview guide was formulated. Next, this guide was tested through internal testing, meaning that pilot interviews were conducted with other researchers from the broader research team (Barriball & While, 1994; Chenail, 2011).^3^ Through this approach, the researcher gained information about the guide itself, such as whether it contained leading questions or ambiguities, and was able to experience the interview as an interviewee. This specific technique of pilot testing is useful when there is limited access to participants (Chenail, 2011), as is the case with elite interviews. During the last phase, the insights gained during pilot testing were incorporated and finalized into a clear and logical semi-structured interview guide. Because the interviews were structured as a guided dialogue, it allowed the interviewer to naturally guide the participants from talking about general ideas on changing behavior in their work settings toward more specific mechanisms on how to do so, and how this would work, without using leading questions. For the goal of this study, it was important that interviewees would mention these mechanisms without any probes or suggestions to truly gather their own ideas. Therefore, the questions were open-ended and the interviewer did not introduce any mechanisms herself. The format also allowed the interviewer to adjust the questions to the flow of the interview and to ask follow-up questions (Brinkmann, 2014). The question guide can be found in “Appendix 1”. Three of the interviewees did not consent to recording; in these instances, the interviewer took notes during and immediately after the interview. One interviewee did not consent to directly use quotes from the interview in the text of this article.

Interviews ranged in length from 35 to 80 min and averaged about 53 min. Because of the COVID-19 pandemic, 20 interviews had to be conducted using video conferencing tools such as Zoom and Microsoft Teams, a suitable technique when face-to-face interviewing is not possible (Gray et al., 2020; Sah et al., 2020). Data elicited via video calls have demonstrated comparable richness as data from face-to-face interviews because factors influencing the quality of data can also be fulfilled online (Keen et al., 2022). Importantly, it is still possible to build participant-researcher rapport online (Roberts et al., 2021). In this research, online rapport was built by using the camera, thanking the participant for their time, checking for technical problems and by asking if there were any questions prior to the interview (cf. Roberts et al., 2021). Moreover, prior to the interview, participants were asked about preference in virtual platform: by using a platform they were familiar with, the risk of technical issues was minimized (Lobe et al., 2020; Roberts et al., 2021). One interview was conducted face-to-face. All online participants received an email with the information sheet and informed consent form prior to the interview. The face-to-face participant received a paper version prior to the start of the interview. One interviewee withdrew consent and was not included in analyses.

### Coding

Although some of the “personal touch” associated with face-to-face interviews was lost in the virtual interviews, I felt that the participants spoke open and honestly about their ideas and perceptions. Therefore, the interviews were considered suitable for data analysis. The data analysis started concurrently with the data collection. After 18 interviews, saturation was reached (cf. Guest et al., 2006). To ensure that no new mechanisms were mentioned, three additional interviews were conducted. As no new mechanisms were mentioned, data collection was stopped.

After transcriptions of the interviews were made, the data were coded and analyzed based on thematic analysis (Clarke & Braun, 2017) using ATLAS.ti 22. No codes existed before analyzing the first interview. First order coding was done open and inductively (Williams & Moser, 2019), and codes were based on the mechanisms mentioned by respondents. The unit of analysis was the individual compliance officer and all interviews were coded in comparable ways (e.g., not all interviews created new codes). The labels of these codes emerged from the interviews, using the language of the participants. All mechanisms mentioned by the interviewee were coded (and in the last stage of analysis counted) to prevent cherry picking or overreliance on specific participants. When new mechanisms emerged, other transcripts were re-examined and prior made codes were refined if needed (Locke et al., 2022). In the next step, the codes referring to all mentioned mechanisms were grouped together in general codes, a form of axial coding and selective coding (Williams & Moser, 2019; see also Saldaña, 2016). These general codes (i.e., the concepts) were globally compared to concepts existing in social scientific literature (Locke et al., 2022). The labels of these codes were based on the concepts mentioned by participants. Last, the interviews were open coded on the level of differentiations made by participants, the perceived effectiveness of the concepts, and other relevant codes that emerged from the interviews. After this coding phase, a number of codes were merged when their concepts were similar to one another (Williams & Moser, 2019). The coding was done by one researcher and was checked through peer-review coding in the different phases with members from the research team (Nowell et al., 2017). A member check was conducted by presenting the findings to a group of Dutch compliance officers in order to ensure the interpretation of the mechanisms mentioned by compliance officers were in line with their intended meaning (Malsch & Salterio, 2016; Nowell et al., 2017). Last, the representativeness of quotes was analyzed by considering the context of the used quotes to ensure that they would not be misleading and obtained free from pressure, and to make sure the quotes are not biased in favor of this study (Eldh et al., 2020; Rockmann & Vough, 2023).

### Reflexive statement

Unintentional researcher influence is inevitable (Schwarz, 2007). It is therefore vital to be explicit about my own positionality so readers can assess to what extent this may have shaped the findings. The researcher [MEK] is a highly educated, Western European, 25–35 year old, female researcher without experience as a compliance officer or related jobs. In conducting this qualitative research, the researcher’s positionality is shaped by her own background, experiences, and relation to the research topic. She held no preconceived opinion about compliance officers, but is critical toward the validity and applicability of social scientific research in practice. The researcher continuously reflected on how the interpretations are shaped by her own perspectives and how this may affect the representation of the research findings, and discussed this with other members of her research team.

## Results

The next section will discuss the analysis of the alignment between compliance officers’ experiential knowledge and scientific knowledge on how to prevent organizational misconduct. As the data are of qualitative nature, any claims that may imply causality or statistical patterns should be understood in this qualitative manner. First, it will answer research question 1: how do compliance officers think they can prevent misconduct in their organization? Second, it will answer to what extent their experiential knowledge is aligned with social scientific insights on compliance on a general conceptual level. The last section will focus on the third research question: to what extent is the experiential knowledge aligned with scientific knowledge on a specific substantive level?

### Compliance Officers’ Perceptions on Behavioral Change

A preliminary question, before looking more closely at the experiential knowledge of compliance officers about how they can prevent misconduct, is whether they even see such prevention and behavioral change as part of their job. All 21 compliance officers mentioned that changing behavior was a crucial part of their job and that compliance as they see it is more than solely complying with rules, laws, and policies: there is a large “gray area” of conduct that is relevant to them. Participants underlined that preventing misconduct is not only about preventing illegal behavior such as fraud, but also about unethical behavior, harassment, and inappropriate conduct.

In order to assess the relevant experiential knowledge of compliance officers, they were asked how they could change behavior within their organization. In response, all participants mentioned multiple concepts through which they believe they can try to change organizational behavior. In total, eight concepts through which compliance officers expect to change behavior were mentioned by more than five out of 21 participants, as shown in Table 2.^4^ Additionally, Table 3 presents an overview of the concepts mentioned per compliance officer.

**Table 2:** Overview of Concepts Mentioned by More Than Five Compliance Officers

Mechanism	Number of participants mentioned
Organizational culture	19
Knowledge of rules	18
Sanctions	18
Role modeling and leadership	17
Discussing rules and behavior	17
Understanding rules	11
System architecture	9
Reporting and whistleblowers	8

**Table 3:** Mentioned Mechanisms per Compliance Officer

	Organizational culture	Knowledge of rules	Sanctions	Role modeling and leadership	Discussing rules and behavior	Understanding rules	System architecture	Reporting
1	X	X	X	X	X	X	X	
2	X	X	X	X			X	X
3	X	X	X	X	X			
4	X	X	X	X	X		X	X
5		X	X	X		X	X	
6	X		X	X	X	X		
7	X	X	X		X	X		
8	X	X	X	X	X			
9	X	X	X	X	X	X		
10	X		X		X		X	
11	X	X	X	X	X	X	X	X
12	X	X	X	X	X	X		
13	X	X	X	X	X		X	
14	X	X	X	X	X	X		
15		X	X	X	X	X		X
16	X	X		X	X			X
17	X	X	X				X	X
18	X		X	X	X			X
19	X	X	X	X	X	X		
20	X	X			X	X	X	
21	X	X		X				X

Tables 2 and 3 thus show that compliance officers have a broad view on what shapes corporate compliance. First, the participants collectively identify a wide range of mechanisms that, according to them, are at play (Table 2). They do not just think that compliance is driven by a small subset of mechanisms or related interventions (such as just fear of sanctions or leadership). Instead, most see an interplay of many different factors. As such, they do not believe there is just one solution, or one quick fix for more compliant behavior and less organizational misconduct. As one participant said: “I think that there is more than one way to Rome. I have multiple strategies, because I think it is too fragile to choose one strategy” (CO019).

We can look more closely at their answers by examining the variation between individual compliance officers. Table 3 shows that compliance officers typically mentioned a larger number of distinct mechanisms. There is also much overlap between the types and number of concepts mentioned by different compliance officers. Almost all compliance officers mentioned organizational culture (19 out of 21), knowledge of rules (18 out of 21), sanctions (18 out of 21), role modeling and leadership (17 out of 21), and discussing rules and behavior (17 out of 21). All interviewed compliance officers mentioned a minimum of four concepts, with most mentioning six different concepts. Thus, based on the qualitative data, one may conclude that it seems that the group of interviewed compliance officers have somewhat similar ideas on how they can change behavior within the organization without substantial individual differences.

### General Conceptual Alignment of Knowledge on Preventing Misconduct

This section will report to what extent the concepts mentioned by compliance officers align with the concepts that are distinguished in the social scientific literature on compliance.

Based on the above section, it seems that compliance officers recognize that multiple concepts are at play, as suggested in research (Kuiper et al., 2023; Weaver, 2014). Thus, there seems to be an alignment in the complexity that both compliance officers and research recognize. In order to analyze the alignment on the conceptual level, Table 4 presents an overview of the concepts mentioned by the compliance officers, as coded inductively, and corresponding concepts in the existing social science literature. An extensive explanation of the views of compliance officers on the other concepts can be found in “Appendix 2”.

**Table 4:** Overview of concepts mentioned by compliance officers and corresponding concepts mentioned in literature

Compliance officers	Literature
Organizational culture	Organizational culture
Knowledge of rules	Legal knowledge
Sanctions	Punishment
Role modeling and leadership	Ethical Leadership
Discussing rules and behavior	
Understanding rules	Legal knowledge/legal uncertainty
System architecture	Nudging and choice architecture
Reporting and whistleblowers	Reporting and whistleblowers

Many of the salient concepts mentioned by compliance officers are also concepts studied in social scientific literature, although the language used may not be exactly the same. Most of the compliance officers discussed how important organizational culture is and mentioned various forms, both positive and negative, of organizational culture. Many different descriptions were given of organizational culture, varying from group behavior to norms and values or even “subordinate levels.” There is no clear definition that derived from the interviews, as participants had difficulties explaining the concept:

To implement it in the culture of the organization; that may be the most difficult thing, to make that intrinsic for people so that it is fully a part of the culture procedure. I think that that is super important but also really complicated. You are above human, more on a system level. (CO017)

Overall, participants described how a good culture would lead to more compliance, and a bad culture could lead to more misconduct. In the literature, there is extensive attention given to organizational culture and its influence on behavior too (De Bruijn, 2021; Kaptein, 2008, 2011; Schwartz, 2013; Treviño et al., 1998; Van Rooij & Fine, 2018).

Eighteen participants described how they thought it is important that employees know the rules that they have to comply with in order to be able to do so:

I think that it always starts with a bit of education about the subject. There is so much within compliance, so many subjects, so much that is already quite well organized. But if you were to discuss this with a random employee who does not work in compliance, I dare say that maybe half of the subjects would be unknown to them. And with that, you have to stimulate that, to repeat that every quarter or year and make people aware of it. (CO009)

The concept of knowledge of legal rules overlaps with the concept of legal knowledge that has been shown to matter for corporate compliance (Van Rooij, 2021).

Eighteen participants described how they thought that sanctions could prevent future misconduct. For example, CO012 said:

But the idea of punishment, of course, is that, on the one hand, you are simply just punished for the thing that you did, but on the other hand, it also prevents an employee from doing that thing again in the future.

The idea of sanctions overlaps with research on punishment in organizations (e.g., Hersel et al., 2019). It also overlaps with research on punishment in a broader context outside of organizations, mostly focusing on criminal legal punishment (van Rooij et al., 2025).

Participants further mentioned how role modeling and leadership could help corporate compliance:

I think that role-modeling behavior is very important. I think that it is important—and I tell this to managers too—that they are aware of the important position that they are in, both as manager of the people themselves and of the process. That they are in the position to create circumstances in which people can function with integrity. That means, among other things, that they have to keep people updated about which rules they have to comply with and what the appropriate manners are. (CO019)

In total, 17 compliance officers described that it would show employees what the expected behavior is. This idea has also been researched. There is research focusing on ethical role models in organizations, which is also referred to as ethical leadership (Brown & Treviño, 2014).

Compliance officers also mentioned that discussing rules and behavior is important for compliance. Participants described how such discussion could clear up gray areas and dilemmas. As CO005 explained:

If you have a question in that area, or the question: ‘How do we interact with each other?’ Or about what you can do with a client—that often involves gray area issues. To have open conversations about that with coworkers. Or to discuss it with the compliance department, if you want to. But it is not weird to have questions about these things, and sometimes things can be a bit gray—the whole concept: dilemmas.

Furthermore, through such discussion, compliance officers can explain rules and expected behavior. Last, it allows them to discuss potential misconduct, giving them more insights into people’s behavior. The idea as described by the participants corresponds with several academic bodies of work: legal knowledge (Van Rooij, 2021), social norms (Cialdini & Goldstein, 2004; Cialdini & Trost, 1998) and ethics training (Hess, 2021).

Similarly, eleven compliance officers explained how it is important that employees understand rules in order to be able to comply with them. One compliance officer explained:

And I think that it is an important part of the training you give. If someone starts working at a company, you should not only explain: ‘These are the rules,’ but particularly why we have these rules and why it is important to behave this way. Just an explanation of the rule itself does not do much. (CO0015)

Although this is not a direct concept within the literature, it overlaps with the ideas of how legal knowledge and uncertainty about the law shape compliance (e.g., Feldman & Teichman, 2009).

Nine participants described how compliance can also be increased through system architecture: internal processes can make it easier for employees to do the right thing and make it more difficult to commit misconduct. One compliance officer mentioned:

Then you will have to implement a change in system or behavior: compliant behavior can also require a systemic change. It is not always behavior. Sometimes, we just need to add a checkbox to the system, or put an extra module in the system, which prevents you from continuing. With that, you can also stimulate compliant behavior. (CO021)

This is in line with the idea of nudging compliance through choice architecture (Kantorowicz-Reznichenko & Wells, 2021) as well as by reducing opportunities for misconduct (Van Rooij & Fine, 2021).

Participants also described that the reporting of violations or complaints could lead to less misconduct, although they did not clearly describe how this would do so. For example, CO017 said: “You want to take reports seriously, take them, investigate them, and five feedback, then draw a conclusion and learn from them.” The idea of reporting overlaps with research about the functioning of whistleblower protection systems (Culiberg & Mihelič, 2017).

All of this shows that, on a general level, there seems to be an alignment between the experiential knowledge of compliance officers and social scientific knowledge. First, they both describe a multitude of concepts that are at play when preventing misconduct. Furthermore, the concepts mentioned by the interviewed compliance officers are also concepts that are researched in relation to compliance. Only the concept of discussing behavior and rules does not directly correspond with a single researched concept, but the idea overlaps with multiple concepts that have received attention from researchers.

### Specific Substantive Alignment Between Scientific and Experiential Knowledge

To gain a better understanding of the alignment, it is important to not only understand the alignment between the concepts that are distinguished by compliance officers and by the science, but also to analyze the alignment between the way that either of them understands these concepts to actually change behavior. Accordingly, it is also necessary to assess the substantive alignment. To be able to make an analysis of the substantive alignment between the ideas of compliance officers and existing literature, the choice of concepts was dependent on multiple conditions. First, in order to give a proper representation of the view of the interviewed compliance officers, I focused on concepts mentioned by more than half of the compliance officers. Therefore, the concepts of understanding rules, system architecture and reporting and whistleblowing (which were mentioned less) were not analyzed. Second, it was important that the interviewees (clearly) explained the concept and how the concept may affect behavior to be able to make the comparison with literature. As respondents did not explicate this for organizational culture, this concept could not be analyzed. Third, it was necessary to focus on concepts on which there is a sufficiently well-developed body of work, in terms of having been extensively studied and reviewed. As the concept of discussing rules and behavior related to multiple bodies of work which formed an obstacle for a clear analysis and the body of work on legal knowledge in light of business compliance is somewhat limited (cf. Van Rooij, 2021), the next section will focus on the substantive alignment between experiential knowledge and scientific knowledge of two concepts: first, role models and leadership, and second, sanctions. A further explanation of the views of compliance officers on the other concepts can be found in “Appendix 2”.

#### Alignment of Knowledge About Sanctions

In total, eighteen compliance officers explained that they believe that with some cases of misconduct, sanctions are a possible way to change behavior and therefore prevent misconduct. One compliance officer explained:

But the idea of course of punishment is that on the one hand, you are simply just punished for the thing you did, but on the other hand also prevents that an employee will do it again in the future. (CO012)

Seventeen compliance officers talked about sanctions on an individual level. When interviewed compliance officers mentioned sanctions, they mostly referred to internal corporate sanctions, such as formal warnings to employees and dismissals. None of the compliance officers thought that sanctioning employees is a suitable first instrument to prevent misconduct in the organization, but all 18 thought it was necessary to have the option.

In the literature, there is extensive research on the broad idea of punishment preventing misconduct, originating from many disciplines. It is important to note that research on the effect of internal organizational sanctions to prevent employee misconduct is rather limited (Treviño & Weaver, 1998). Most academic studies that focus on punishment in the organizational setting have focused on the effect of external administrative or criminal sanctions such as fines or imprisonment on corporate compliance (e.g., Schell‐Busey et al., 2016). A much smaller body of work has looked at internal sanctions and informal sanctioning processes such as leaders’ disapproval as a form of punishment (e.g., Podsakoff et al., 2006). Besides the literature that specifically focuses on the organizational context, there are also informative findings in the broader literature on punishment, mostly focusing on the different effects that punishment may have on criminal behavior generally, both in a positive and negative way (van Rooij et al., 2025).

Compliance officers distinguished two mechanisms through which they believed sanctions can mitigate future misconduct that are also acknowledged in the literature. First, four compliance officers stated that sanctions can have a deterrent effect. In particular, firing someone or sanctioning an individual in a management position could have a deterrent effect. CO011 explained:

Therefore, you have to use people as an example, because that has to serve as a deterrent to prevent others from doing that thing. Those kinds of people have to be banned for life from such positions, that sort of stuff.

Conversely, four compliance officers doubted whether sanctions have a deterrent effect. The participants mentioned specific conditions under which they thought the sanction would be effective in preventing future misconduct. One compliance officer elaborated that the deterrent effect is not only about the severity of the punishment, but rather that the certainty of punishment is more important. Five participants pointed out that the effect of sanctions depends on the individual receiving the sanction: for some individuals, a warning will be enough; others will always try to find a way to continue the misconduct. Last, five compliance officers thought that it is important to be transparent and open about the behavior and the subsequent sanction to other employees. However, four compliance officers pointed out that this is challenging because of privacy issues. CO012 explained:

But we have situations that are difficult, for example when an employee has shown inappropriate behavior and that is the cause of firing them or dissolving the contract. The difficulty is that, because of privacy issues, you cannot communicate much about it to other employees, and that can give them the feeling of: ‘You can simply just disappear here.’ That is a fine line that you are on.

In empirical research, there has been much attention given to the deterrent effect of punishment. In line with the views of the compliance officers who doubted this effect, Schell‐Busey et al. (2016) did not find conclusive evidence in their meta-analysis that punitive sanctions have a deterrent effect on individual-level corporate offending. It is important to note, however, that this review focused on administrative and criminal punishment, and not on internal sanctions within the organization. In general literature focusing on traditional crimes, we also see inconclusive evidence for a deterrent effect of punishment (Chalfin & McCrary, 2017; Nagin, 2013; Petrich et al., 2021).

Research has also focused on the conditions under which a deterrent effect may occur. Overall, the conclusion is that certainty of punishment is more important than the severity of punishment (Brown, 1978; Chamlin, 1991; Nagin, 2013), which is in line with what one compliance officer stated. Furthermore, the idea that the effect is dependent on the individual is also reflected in the literature: work on deterrability suggests that not everyone can be deterred through punishment (Jacobs, 2010). Here, the idea is that one group of people is deterrable with punishment, one group is not because they would never commit a crime, even in the absence of punishment, and one group is not deterrable because of individual characteristics that limit their capacity to engage in deliberate thoughts (Herman & Pogarsky, 2022; Jacobs, 2010; Pogarsky, 2002).

Last, the idea that the sanction has to be clearly communicated in order to be effective aligns with the idea that the perception of punishment is important. Research shows that the subjective perception of the threat of punishment is crucial: a clear communication about the threat of sanctions is important (Apel, 2022; Apel & Nagin, 2017; Waldo & Chiricos, 1972).

As such, we see a partial alignment in the ideas about deterrence between compliance officers and scientific research: whereas some compliance officers’ views resemble the state of scientific knowledge (that there is inconclusive evidence about the deterrent effect of sanctions), others were more convinced about this effect than scientific findings can confirm. Additionally, both compliance officers and research distinguish conditions under which punishment may or may not be effective in preventing future crime. At the same time, an important caveat to these findings is that internal sanctions (such as disciplinary actions) have not been studied much in the empirical literature. As such, more research on this subject is needed in order to understand whether compliance officers’ ideas would align with more tailored empirical evidence about this question.

The second mechanism through which compliance officers believed sanctions can prevent misconduct is through norms: four participants described that they think a sanction can help to set a norm as it communicates what appropriate behavior is. The sanctions provide a clear line between wanted and unwanted behavior. CO015 said: “I think a bit of clarity; on a certain level punishment makes it clear what is appropriate behavior and what is inappropriate behavior. Maybe that is the most important reason for having sanctions.” If misconduct is not followed by sanctions, it can also give the impression that the misconduct is the accepted norm:

Also, I believe that things that are really bad have to be punished. Those are things that are unacceptable. If you see that those things are not handled strictly, it leaves space for others to act on that. They will think: ‘Nothing will happen; I can do this too.’ I think that those sorts of things should be mentioned clearly by management and not be covered up. (CO009)

Most of the literature on how law can communicate norms (e.g., Van Hoecke, 2002) has not empirically studied how punishment plays a role in setting such norms (van Rooij et al., 2025). There has been some work in the organizational context on the effects of a lack of punishment, meaning law would fail at communicating norms, but this work has predominantly been normative rather than empirical (Pontell et al., 2014; Steinzor, 2015). Here, these scholars argue that white-collar criminals should be prosecuted in order for law to successfully communicate norms, without showing empirical evidence about how such communication works and how it affects misconduct or rule breaking.

Compliance officers believed that sanctions within the organization can also have negative consequences. Five compliance officers described how sanctions can have negative consequences, especially when there is a lack of transparency. A lack of transparency can happen because of privacy reasons, when a compliance officer is unable to share the details of a sanction or misconduct. This can lead to rumors or people believing that the punishment is unjust or too harsh. The participants described how overly harsh or frequent sanctions can lead to an unsafe culture on an organizational level, possibly leading to more misconduct: “If they think that there is very severe punishment with respect to what the employee did, that can really elicit feelings of unsafety” (CO012). Research found that punishment that is perceived to be unjust in the workplace can cause more misconduct (Mooijman & Graham, 2018). Additionally, research also suggests that punishment could lead to fear in the workplace, with the result that people are afraid to speak up (Kish-Gephart et al., 2009). However, there is no clear empirical evidence on the relation as described by practitioners, that fear caused by sanctions will lead to more misconduct.

The broader literature on punishment shows a broader set of punishment mechanisms than those mentioned by compliance officers (for a review, see van Rooij et al., 2025). For example, research also describes how punishment can have an incapacitation effect, meaning that the punishment makes it practically impossible for the offender to reoffend (Heaton et al., 2017; Piquero & Blumstein, 2007). Firing employees or transferring someone to another department could be seen as a form of incapacitation, but it was not explicitly described as such by participants.

In sum, there is some alignment between the ideas of practitioners and literature: there is an overlap in the distinguished mechanisms through which punishment could prevent misconduct. Both compliance officers and scientific research distinguish both positive and negative effects of punishment on misconduct. At the same time, we can conclude that the participants focus on possible processes such as communicating norms or instilling fear that have received less empirical attention in the literature. On the other hand, we see that in the research, more mechanisms are distinguished by which punishment may affect behavior than mentioned by the participants. However, more research is needed to fully understand the influence of sanctions in the workplace on individual organizational misconduct and the broader consequences for the organization.

#### Alignment of Knowledge About Role Models and Leadership

The second concept that will be analyzed to research the substantive alignment between compliance officers’ experiential knowledge and scientific knowledge is the influence of role models and leadership on corporate compliance. In total, 18 compliance officers described how important leadership is for behavioral change, and especially role modeling from leaders. Compliance officers think that role modeling and (good) leadership can lead to less organizational misconduct. Nine participants mentioned the term “tone at the top” in relation to role modeling and leadership, meaning that the tone at the (highest) management levels should show what the correct behavior is. Three of them added the term “tone at the middle.” For them, it is equally or even more important that not only the absolute top, such as a CEO, but also middle management act as role models for their teams:

So not only top management—it is an important precondition that is often also an audit requirement that you can show that top management says things about integrity—but especially that combination with local management is what really elicits behavioral change. (CO006)

Participants described two aspects that are important for role modeling and leadership. First, participants elaborated on how they think leaders and managers have to be clear about what is important within the organization. Seven compliance officers think that a manager has to communicate what is allowed and what is not:

I think that role-modeling behavior is very important. I think that it is important—and I tell this to managers too—that they are aware of the important position that they are in, both as manager of the people themselves and of the process. That they are in the position to create circumstances in which people can function with integrity. That means, among other things, that they have to keep people updated about which rules they have to comply with and what the appropriate manners are. (CO019)

Additionally, five participants said that it is important that leaders convey a clear message about compliance.

Second, participants emphasized that the actual behavior of leaders matters. In total, more than half of the participants said that managers have to behave as role models, meaning that they have to demonstrate compliant and ethical behavior. This behavior also has to match the message communicated by leaders. One participant emphasized that leaders’ behavior must be visible within the organization. For five compliance officers, it is important that managers are an example in being open: they have to be open about their own mistakes, doubts, and dilemmas. This will set the example for other employees to do the same. Consequentially, they will discuss their dilemmas and mistakes more easily and learn from them. This will prevent future misconduct and contribute to a safe work environment.

Thus, compliance officers mostly describe how role modeling and leadership can have positive consequences as it can prevent misconduct. Only one participant focused on the possible negative effects of showing unethical behavior as a role model:

Literature shows that when directors, managers, and other important people in an organization do not show the correct behavior, it is also easy for other employees to use this as an excuse to behave with less integrity and to make less ethical considerations. (CO019)

In the broader literature in business ethics, organizational science, and organizational psychology, scholars have also looked at different aspects of role modeling and leadership. To assess the alignment between this scientific body of work and the experiential knowledge of compliance officers, I will compare what the officers described, as outlined just above, with relevant similar topics studied in empirical work.^5^

Similar to the compliance officers, scholars also find that leadership is an important factor for ethical and unethical behavior inside organizations (Bedi et al., 2016; Brown & Mitchell, 2010; Brown & Treviño, 2006). Here, the concept of role modeling and leadership is often referred to as ethical leadership (Den Hartog, 2015). Ethical leadership has been defined as “the demonstration of normatively appropriate conduct through personal actions and interpersonal relationships, and the promotion of such conduct to followers through two-way communication, reinforcement, and decision-making” (Brown et al., 2005, p. 120). The idea is that employees will come to behave similarly to their leader through imitation and observational learning, in line with Bandura’s classic social learning theory (Bandura, 1986; Bandura & Walters, 1977). The premise of the theory is that people look to role models for behavioral cues and guidance. Leaders in organizations are salient role models for employees because of their power and status. Additionally, ethical leadership has been studied in terms of social exchange (Cook et al., 2013). Here, the idea is that when employees are treated ethically by their leaders, they will reciprocate (e.g., Hansen et al., 2013; Hassan et al., 2013). The process as described by compliance officers seems more in line with social learning theory than with social exchange.

Interviewed compliance officers believed that good leadership and role modeling will lead to less misconduct. Research has indeed related ethical leadership to employee behavior. Most research has focused on positive behavioral outcomes, such as prosocial helping behavior (e.g., Mayer et al., 2009) or job performance (Piccolo et al., 2010). Other research has found a negative relation between ethical leadership and employee deviance and unethical behavior (Avey et al., 2011; Brown & Treviño, 2006; Mayer et al., 2009, 2010, 2012; Neves & Story, 2015).

A term used by participants in relation to role modeling and leadership was “tone at the top.” Management and business ethics literature has also focused on this concept as part of their focus on ethical leadership (Lail et al., 2015; Pickerd et al., 2015; Treviño et al., 2008; Warren et al., 2015). In such literature, tone at the top is described as “top management’s way to express (ethical) values pursued in the organization and provide guidance to employees” (Ewelt-Knauer et al., 2022, p. 610), although definitions can slightly differ between researchers (Warren et al., 2015). Tone at the top is a precursor to or reinforcement of ethical culture or ethical norms (Ewelt-Knauer et al., 2022). The idea of participants that the concept of tone at the top is a predictor of ethical behavior is in line with research findings (Warren et al., 2015). However, Schaubroeck et al. (2012) found that the values of management are not always adopted by employees. One explanation of how tone at the top may influence employee behavior is through social norms (Ewelt-Knauer et al., 2022). Broader previous research has found that social norms can lead to changes in behavior, resulting in more compliance (Cialdini & Goldstein, 2004; Cialdini et al., 2006; Goldstein et al., 2008; Schultz et al., 2007). Ewelt-Knauer et al. (2022) concluded that social norms related to tone at the top are also linked to organizational misconduct.

Although most participants focused on how role modeling and leadership have positive effects, one participant emphasized that leaders showing unethical behavior as an example could have negative effects. Indeed, research has found support that unethical behavior from leaders can lead to more unethical behavior in the workplace (Brown & Mitchell, 2010; Hassan et al., 2023; Kemper, 1965; Treviño & Brown, 2005). Research also suggests that not only can unethical examples from leaders lead to more misconduct, but also that role modeling and ethical leadership in general can have possible negative (unintended) effects in organizations. Heres (2016) concludes, in her study on the ethical leadership of high government officials, that small mistakes are easily made and easily noted, but not easily forgotten by employees. Others found that leaders who are perceived as excessively ethical may be less attractive role models (Stouten et al., 2013). Research has also shown some pitfalls regarding tone at the top. First, it is often unclear exactly who the top is (Warren et al., 2015). In practice, there can be multiple tops on multiple levels. Research shows that different (groups of) leaders risk communicating conflicting tones, which can have unintended negative effects. Pickerd et al. (2015) found that conflicts in tone can lead to more organizational misbehavior. Such unintended effects caused by conflicting tones or leadership in general were not specifically mentioned by participants: they were less clear about possible negative effects. Although there is some indication that ethical leadership can possibly have negative consequences, more research is needed.

In conclusion, we see a superficial alignment between the experiential knowledge of compliance officers on role modeling and leadership and the relevant body of scientific work. Both emphasize the importance of good role modeling and leadership for ethical behavior within the organization. However, zooming in on what this actually means, there is more misalignment. The interviewed compliance officers tend to talk about this relation in a causal way (good leadership will lead to less misconduct), while the research is more limited to correlations between leadership and employee behavior (there is a relation between the two). Furthermore, the participants elaborate less on *how* they believe the process works, whereas scientific research is based more on theory building, aiming to understand the processes. Last, the compliance officers tend to focus less on the conditions that are needed for good role modeling and leadership. In other words, they tend to focus less on possible negative effects, contrary to the body of research. In fact, research suggests that the relation between leadership and employee behavior is more complex than is suggested by the compliance officers.

## Discussion

Compliance officers play a crucial role in preventing organizational misconduct. So far, however, only limited research has been done on this profession (Den Nieuwenboer et al., 2025). Studying what compliance officers think and how they do their work is useful for the field of business ethics to further understand ethics in practice: compliance officers play a key role in how business manage ethics (Den Nieuwenboer et al., 2025). This study aimed to add to the existing literature on compliance officers, providing more insight on their experiential knowledge: how do they think they can prevent misconduct? The second goal was to research whether the experiential knowledge of compliance officers is aligned with scientific research on organizational compliance. It is important to emphasize the small sample of this study. This means that the results cannot be generalized to a broader sample. Instead, these findings can help us to increase our understanding of compliance officers and their views on appropriate methods to mitigate misconduct, and how the answers of the interviewed sample align with empirical research.

The first finding of this research concerns the experiential knowledge of compliance officers. The results shows that the interviewed compliance officers distinguish multiple mechanisms for changing organizational behavior. Compliance officers do not believe in a quick fix through one solution to enhance compliance but rather rely on a combination of multiple mechanisms. All compliance officers mentioned multiple mechanisms through which they believe they can change behavior within their organization.

On a conceptual level, it seems that there is overlap between the interviewed compliance officers. It is quite interesting that these compliance officers seem to have a level of agreement on what types of mechanisms, and how many, are at play in corporate compliance. The compliance officers interviewed do not work in one organization, yet they seem to share similar thoughts. One possible explanation for this could be that compliance officers nevertheless are connected to each other. Multiple compliance officers mentioned that they create networks with other compliance officers, including those from different organizations. The goals of such networks are to share knowledge, discuss difficult situations or dilemmas, and learn from other organizations. Another possible explanation could be that, although the majority of compliance officers were trained in law, more than 50% stated that they had received some sort of additional training in behavioral change related to compliance. More than 80% of the sample either received such training or studied social science.

Due to the limited sample, the data did not readily allow to compare subgroups in the data on what they said about these mechanisms. Some minimal differences between subgroups seem to emerge, such as that reporting was mostly mentioned by compliance officers working in the public sector (6 interviewees), and only by 2 out of 13 compliance officers working in the private sector. Importantly, due to the qualitative nature of this data, no causal claims or strict statistical associational claims can be made about these observations. Larger patterns, for example based on years of experience, education or gender, could not be discovered. Future research is needed to further develop our understanding of possible patterns and differences between compliance officers and their experiential knowledge.

The second finding of this research concerns the alignment between experiential knowledge and social scientific research. The results indicate that on a general conceptual level, we see that there is overlap in the concepts: most concepts mentioned by the compliance officers have also been researched in relation to organizational compliance.

To analyze the alignment on a substantive level, I compared the knowledge on the concepts of “sanctions” and “role modeling and leadership.” Here, it seems that there is an alignment on a superficial level: both practice and research have a similar understanding of the concepts and see similar possible effects. However, the compliance officers only minimally elaborated on *how* they thought the concepts may affect behavior. This has received more focus in research. For example, research on punishment distinguishes more possible mechanisms through which punishment may affect behavior. Additionally, for role modeling and leadership, compliance officers tend to focus less on the conditions needed to get positive outcomes, overlooking possible negative (unintended) effects. Thus, we see that compliance officers look at behavioral change on a more superficial level than empirical research. It could be risky for compliance officers not to think deeper about how concepts may affect behavior, as they may overlook unintended negative effects. We see this, for example, for role models and leadership: the misalignment here is that science acknowledges possible negative consequences, whereas only one compliance officer mentioned this. On this substantive level, there is opportunity for better communication between empirical research and practice. This could also stimulate compliance officers to ask deeper questions about how concepts may or may not affect organizational behavior to enhance their understanding of this.

To simply conclude that compliance officers’ experiential knowledge and social scientific knowledge on organizational compliance and preventing misconduct are roughly aligned is risky. As stated before, the sample of this research is small: these are preliminary findings about the interviewed compliance officers. First, it could be that the use of certain concepts can have unintended negative consequences that compliance officers do not always see. Second, the current body of research is not sufficiently developed to answer questions about the effectiveness of concepts for minimizing organizational misconduct. The empirical study of corporate compliance has inherent trade-offs (Van Rooij & Rorie, 2022). First, accurately measuring compliance and illegal behavior is challenging, as people may not answer questions honestly and it also presents major ethical challenges to do so. Second, research on corporate compliance often encounters difficulties establishing a clear causal relationship that also translates outside of the research setting. Additionally, on some concepts, there is not a large enough body of empirical work that can immediately support practice. A good example is research on sanctions in organizational settings: little to no research has focused on the relation that is salient for compliance officers, namely that internal sanctions can lead to less misconduct (Treviño & Weaver, 1998). Instead, research tends to focus on administrative or criminal external individual sanctions, or general criminal behavior. Moreover, compliance officers do not only focus on illegal behavior but on unethical behavior in a broader sense. In research, there has been much less attention given to how sanctions affect unethical behavior, as the focus is mostly on corporate crime (Greve et al., 2010). Although existing research about punishment can be informative, the question remains as to whether this is directly translatable to this specific setting of internal sanctions in organizations.

This study reflects the experiences of compliance officers: there is no quick fix toward more compliant behavior within companies, although sometimes management may expect such fix from compliance officers (Soltes, 2021). In their view, it is a complex interplay different mechanisms influencing behavior (cf. Kuiper et al., 2023; Weaver, 2014). At the same time, this study hints toward a possible blind spot: the possible negative consequences of mechanisms to influence behavior. Not all efforts result in positive change, they can also result in more misconduct. It is important that compliance officers, management, and all others responsible for preventing misconduct become aware of such blind spots in order to be actually effective in preventing such misconduct. For example, ethical leadership is becoming increasingly important (and popular) within organizations (Amory et al., 2024), but this study shows that most compliance officers in this sample are not aware of its possible downsides. Thus, this carries the risk that compliance officers may adopt ethics interventions that look right, but fail to address the actual underlying behavioral mechanisms, or may even cause harm. Scientific information could help to raise awareness of such blind spots. Further research is needed to gain a better understanding of such blind spots.

A noteworthy encouraging insight may be that compliance officers already see compliance as a complex system of mechanisms and reject quick fixes, meaning that they may be open to integrate richer, evidence-informed ethical and behavioral insights into their strategies. This could enhance the collaboration between practice and research: compliance officers or managers can play a key role in implementing evidence-informed strategies, or can work on evaluating what works in practice, where researchers can facilitate this process by co-creating interventions with practitioners, or aim to translate abstract theoretical insights into practice tools. Together, such efforts can enhance the integration of evidence-informed knowledge into organizational practices.

As this was a first study on the experiential knowledge of compliance officers, more research is needed. Further research can focus on other compliance officers, for example from different sectors, different jurisdictions, or different cultural backgrounds. More research will allow to work toward more generalization and a better understanding of the beliefs of compliance officers on how to mitigate misconduct, and how this may align with scientific findings.

### Limitations

This study is not without limitations. First, it is important to highlight that due to the qualitative nature of this study, the findings cannot be generalized to all compliance officers. The current sample reflects a variety of compliance officers, as it includes compliance officers working at different organizations, in different sectors, and both in the public and private sector, but the goal of this paper was not to gather generalizable data. Further research is needed to increase our understanding of the views of compliance officers on mitigating organizational misconduct.

A second limitation is that, in this research, I only focused on what compliance officers think about achieving behavioral change. Compliance officers have more goals in their work than only behavioral change, such as liability management (Soltes, 2021). In other words, compliance officers cannot only focus on effectiveness. Future research should focus on how the different goals relate to each other and what it means for the mentioned concepts.

Third, this study did not allow to analyze additional patterns that may explain possible differences in experiential knowledge between compliance officers. For example, the research did not gather any data on the age or exact years of experience of interviewees, as it was outside the scope of this study. Such data could be insightful to gain a deeper understanding of the nature of the interviewees’ experiential knowledge, and how this may change over time. Future research should include such data to allow for a deeper understanding and to further understand possible differences between compliance officers. Furthermore, future research could investigate possible differences in experiential knowledge by looking at other factors such as education or gender to deepen the understanding of differences and possible patterns.

As noted by one of the reviewers, there is a risk that this research suffers from endogeneity. This is a bias in research, often discussed in the field of econometrics, that addresses the problem of a correlation between the explanatory variables and the error term in a regression (Roberts & Whited, 2013). In the case of this research, endogeneity can be a risk as part of the scientific research on the effectiveness of corporate policies is (partially) based on information from the individuals researched: in-house compliance officers. Therefore, some overlap between the thought of compliance officers and studies about corporate policy may be expected. Nonetheless, not all empirical research used in the analysis is based on work in organizational practice, such as the work on deterrence. Moreover, this research only found an overlap in ideas on a conceptual level, not on a more substantive level. Therefore, it seems that compliance officers still have their own ideas that are not necessarily connected to research findings. Future research could further explore how researchers are shaped by practice in the operationalization of their research.

Last, the quality of the research did not allow for an analysis of the substantive alignment for all mentioned concepts. On the one hand, the fragmentation of findings complicates the possibility of creating a comprehensive overview of scientific evidence. On the other hand, for many concepts, the results are inconclusive or show a complex image (e.g., Hess, 2021; Parker & Nielsen, 2009a). This could also create a barrier for practitioners to actually using scientific findings in practice (Kuiper et al., 2025). These limitations of the current body of work on organizational compliance do not mean that science should be ignored. Rather, it should be the inspiration for enhancing the connection between practice and research to work collectively toward a better understanding of how compliance officers can prevent misconduct and enhance ethical behavior.

## Conclusion

Compliance officers have the important responsibility of preventing organizational misconduct and major scandals with great societal consequences. This study aimed to provide a first understanding on the ideas and beliefs of compliance officers on how to mitigate organizational misconduct. It shows that the interviewed compliance officers believe that there is no quick fix. Furthermore, this study shows that the ideas of the interviewed compliance officers on how to do this are broadly aligned with scientific evidence on a conceptual level, but on a more substantial level we only see superficial alignment.

## Data Availability

Data cannot be shared openly to protect study participant privacy and identity.

## Appendix 1

This question guide contains questions and possible alternatives to ask, depending on the interview and responses of the interviewee.

### Introduction

- Have you always wanted to practice law?
  - Have you always wanted to be a lawyer?
  - What made you decide to become a lawyer?
- Have you always wanted to be a [compliance officer]?
  - Why did you become a [compliance officer]?
  - Why did you choose to become a [compliance officer]?
- Where did you go to law school?
  - What did you study?
  - Tell me about your educational background.

### Behavioral Goals

What are your main goals within your job? [Nudging their thinking toward behavior]

- What in the end are you trying to achieve?
- What in the end is your job about?
- What in the end is your main goal?
- What do you try to achieve?
- How do you see that [compliance officers] are generally successful?
- What is your job about?
- Why do we have [compliance officers]?
  - Why do you think your job matters (for people out there)?
  - Why is it important to have [compliance officers] in society?
  - What kind of societal issues do you try to address in your work?

*Summary of behavioral goal [nudging]*: What I heard you say is that you want to change behavior [prevent organizational misconduct/prevent misconduct/behavioral goal]?

What are the possible ways to [prevent organizational misconduct/prevent misconduct/behavioral goal]?

- How do you achieve this?
  - How do you do this?

What do you mean by [mechanism]?

How does [mechanism] work?

- Can you explain how this works?
  - How does this work to [prevent organizational misconduct/prevent misconduct/behavioral goal]?
  - Why does this [prevent organizational misconduct/prevent misconduct/behavioral goal]?
  - How can this [prevent organizational misconduct/prevent misconduct/behavioral goal]?
    - Any examples?
    - What are the effects of [mechanism]?
    - Can you think of a specific case where you did this?
      - Did this lead to a change in behavior?
      - Why did this work?
        - [Explanation of how the mechanism works]?
- *IF* “I don’t know”
  - Reassurance, then:
    - How do you think this works?
    - What do you think is at play here?
    - If you had to guess, what do you think is going on here?

### Feedback Loop

I also hear you say that you use [mechanism] to [prevent organizational misconduct/prevent misconduct/behavioral goal]?

Are there other ways you [prevent organizational misconduct/prevent misconduct/behavioral goal]?

- How do you achieve this?
  - How do you do this?
- What does this look like in practice?
- What do you mean by [mechanism]?

How does [mechanism] work?

- Can you explain how this works?
  - How does this work to [prevent organizational misconduct/prevent misconduct/behavioral goal]?
  - Why does this [prevent organizational misconduct/prevent misconduct/behavioral goal]?
  - How can this [prevent organizational misconduct/prevent misconduct/behavioral goal]?
    - Any examples?
    - What are the effects of [mechanism]?
    - Can you think of a specific case where you did this?
      - Did this lead to a change in behavior?
      - Why did this work?
        - [Explanation of how the mechanism works]?
  - IF “I don’t know”
    - Reassurance, then:
      - How do you think this works?
      - What do you think is at play here?
      - If you had to guess, what do you think is going on here?

*New topics* (possible short summary of above, not end summary).

- [Thinking beyond the constraints,] any innovative methods that can be tried to prevent lawbreaking?
  - If you were in charge, what would you prioritize?
  - If there were no limitations, what would you do?

### End of Interview

We discussed how to encourage compliant behavior today. You shared your insights, experience and interesting examples. They are very helpful. Anything else you would like to add?

## Appendix 2

### Organizational Culture

Nineteen compliance officers mentioned organizational culture as an important concept to prevent misconduct. The compliance officers described positive and negative forms of organizational culture. Many different descriptions were given of organizational culture, varying from group behavior to norms and values or even “superordinate level.” There is no clear definition that derived from the interviews, as participants had difficulties explaining the concept: To implement it in the culture of the organization; that may be the most difficult thing, to make that intrinsic for people so that it is fully a part of the culture procedure. I think that that is super important but also really complicated. You are above human, more on a system level. (CO017)

Sixteen participants mentioned the importance of a positive culture to influence behavior: “And if you steer behavior, the compliance will come when the culture is right; that is my starting point” (CO001). The most described positive culture is where employees feel open to discuss dilemmas, are not afraid to make mistakes, and feel free to point out each other’s behavior, which will eventually lead to less misconduct. Compliance officers referred to this as an open culture, a speak up culture, a safe culture, or a learning culture.

Six compliance officers described a negative culture and its possible consequences, also referred to as an unsafe culture or a culture of fear. As CO008 said: “If the culture is not right, then you can have all the rules, but they will not have any effect.” A negative culture is described as the opposite of an open culture, meaning that people keep things hidden, taking away the opportunity of creating a learning organization. A negative culture can start with an individual or with something small but can have a “ripple effect,” leading to more organizational misconduct (CO012).

Three compliance officers indicated that they actively measured the culture in their organization or had it measured in the past to keep track of any possible developments. One compliance officer expressed their concerns about the consultancy and business attached to organizational culture at the moment: What you see is that organizations are very busy with big procedures concerning change and ‘we will bring the culture to a new level,’ but I think: what is the culture you are talking about? What is the desired culture? That is difficult ... difficult. I think: what are you going to change? If you cannot make clear where you want to go, what are you going to change? And you see that organizations kind of lose themselves in the number of change procedures, of which I think: now make it clear what exactly it is that you want to change. (CO021)

Thus, although most compliance officers talked about organizational culture, they did not provide a clear definition, nor a clear description, of how they think that organizational culture will prevent future misconduct.

### Knowledge of Rules

In total, 18 compliance officers talked about knowledge of rules as a possible mechanism to prevent organizational misconduct. For these compliance officers, it is important that employees know the rules they have to comply with in order to be able to do so. The participants mentioned that they feel responsible for communicating the rules, educating employees on these rules, and creating awareness for them. CO009 explained: I think that it always starts with a bit of education about the subject. There is so much within compliance, so many subjects, so much that is already quite well organized. But if you were to discuss this with a random employee who does not work in compliance, I dare say that maybe half of the subjects would be unknown to them. And with that, you have to stimulate that, to repeat that every quarter or year and make people aware of it.

The result of employees knowing the rules is, according to the participants, that they know what is expected from them and that it is clear what that entails: there is a shared set of norms and values. This also prevents employees from saying that they did not know the rules after something went wrong. Three compliance officers also aim to create awareness of difficult situations, so that employees will recognize these difficult situations and ask for advice if necessary, or they will report the situations. Nine of the compliance officers mentioned that an important and straightforward way to enhance knowledge is through having a code of conduct. Interestingly, 15 compliance officers mentioned having a code of conduct as part of their compliance program to prevent misconduct, but only these 9 emphasized that people should know the content of the code of conduct. One compliance officer was more skeptical about the code of conduct: Every organization has a code of conduct, for example. But I have never seen a code of conduct that does not say that you have to act professionally, or that you need to have respect, or that the customer is number one. So, all these codes of conduct are the same. Yet, still things go wrong. (CO007)

Compliance officers see multiple ways to enhance knowledge of rules within the organization. The most mentioned way to do so is through training, either online with e-learning or games, or in real life. These trainings also allow compliance officers to get specific knowledge to targeted groups in the organization. However, the interviewees are unsure how effective this really is. Although some mentioned that at least repetition was important for effectiveness, others were more skeptical: “Yes, I am a little bit skeptical about that. I think having some sort of audit to show: ‘Look, we have continuous education for our employees,’ is a ‘must have’” (CO010). Another way to enhance knowledge is by discussing it, either individually or in teams.

Of the 18 compliance officers that explicitly mentioned that knowledge of rules is important for more compliant behavior, 11 mentioned that only knowledge is not enough. For them, it is more important that employees also understand the rules. This will be discussed in more detail under “understanding the rules.”

### Sanctioning

Eighteen compliance officers answered that they consider sanctioning a possible way to change behavior. One compliance officer explained: But the idea of punishment, of course, is that, on the one hand, you are simply just punished for the thing that you did, but on the other hand, it also prevents an employee from doing that thing again in the future. (CO012)

Seventeen compliance officers talked about sanctions on an individual level; three also mentioned sanctions on an organizational level, for example by a regulator. In this section, we will only focus on the individual level. The most mentioned sanction on the individual level was being discharged, by nine compliance officers. Furthermore, warnings and transfer to another team were mentioned as sanctions. None of the compliance officers thought that sanctioning employees is a suitable first step toward preventing misconduct in the organization, but all thought it was necessary to have the option.

The compliance officers saw different mechanisms through which sanctions can change behavior. Four compliance officers stated that sanctions can have a deterrent effect. In particular, firing someone or sanctioning an individual in a management position could have a deterrent effect. CO011 explained: Therefore, you have to use people as an example, because that has to serve as a deterrent to prevent others from doing that thing. Those kinds of people have to be banned for life from such positions, that sort of thing.

One compliance officer elaborated that the deterrent effect is not only about the severity of punishment, but that the certainty of punishment is more important. Conversely, four compliance officers doubted whether sanctions have such an effect, with one even starting to doubt the effect during the interview.

Besides having a deterrent effect, sanctions can also set a norm within the organization, according to four compliance officers. The sanctions provide a clear line between wanted and unwanted behavior. CO015 said: “I think a bit of clarity; on a certain level punishment makes it clear what is appropriate behavior and what is inappropriate behavior. Maybe that is the most important reason for having sanctions.” If misconduct is not followed by sanctions, it can also give the impression that the misconduct is the accepted norm: Also, I believe that things that are really bad have to be punished. Those are things that are unacceptable. If you see that those are not handled strictly, it leaves space for others to act on that. They will think: ‘Nothing will happen; I can do this too.’ I think that those sorts of things should be mentioned clearly by management and not be covered up. (CO009)

Whether sanctions will have such a positive effect, resulting in less misconduct, is dependent on multiple conditions. Five compliance officers point out that this is dependent on the individual receiving a sanction: for some individuals, only a warning will be enough; others will always try to find a way to continue the misconduct. Furthermore, five compliance officers thought that it is important to be transparent and open about the behavior and the subsequent sanction with other employees. However, four compliance officers point out that this is challenging because of privacy. CO012 explained: But we have situations that are difficult, for example when an employee has shown inappropriate behavior and that is the cause of firing them or dissolving the contract. The difficulty is that, because of privacy issues, you cannot communicate much about it to other employees, and that can give them the feeling of: ‘You can simply just disappear here.’ That is a fine line that you are on.

Along the same lines as what CO012 described, another four compliance officers also see how sanctions can have negative consequences, especially when there is a lack of transparency. Too harsh or too many sanctions can lead to an unsafe culture on an organizational level, possibly leading to more misconduct: “If they think there is very severe punishment with respect to what the employee did, that can really elicit feelings of unsafety” (CO012).

### Role Models and Leadership

In total, 17 compliance officers described how important leadership is for behavioral change, and especially role modeling from leaders. Nine compliance officers used the term “tone at the top” to refer to this, meaning that top management needs to serve as role models for the rest of the organization. Three of them added the term “tone at the middle.” For them, it is equally or even more important that not only the absolute top, such as a CEO, but also middle management act as role models for their teams: So not only top management—that is an important precondition that is often also an audit requirement that you can show that top management says things about integrity—but especially that combination with local management is what really elicits behavioral change. (CO006)

The other eight compliance officers talked about management in general as role models and good leaders. Two COs added that not all role models have to be leaders or managers; all employees can be role models. This would show others that it is possible for everyone to comply.

In general, leaders and managers have to be clear about what is important within the organization: Seven compliance officers think that a manager has to communicate what is allowed and what is not: I think that role-modeling behavior is very important. I think that it is important—and I tell this to managers too—that they are aware of the important position that they are in, both as manager of the people themselves and of the process. That they are in the position to create circumstances in which people can function with integrity. That means, among other things, that they have to keep people updated about which rules they have to comply with and what the appropriate manners are. (CO019)

According to three compliance officers, leaders have a great impact on the organizational culture: “So how do you achieve such a safe culture? That is really dependent on leadership. On all levels, right. From the team leader, to the front man, to the directional board.” Leaders and management have to be aware of the influence they can have on the culture within the organization, and act upon it.

The compliance officers mentioned different subjects in which management has to be a role model to eventually lead to less misconduct. The first step in being a role model as manager is, for five compliance officers, to have a clear message about compliance. But at the same time, their behavior has to match that message. More than half of the participants said that managers have to behave as role models, meaning that they have to demonstrate compliant and ethical behavior. One compliance officer emphasized that this behavior of leaders has to be visible within the organization. The idea is that if managers show ethical behavior, employees will follow and also show this behavior. One compliance officer focused on avoiding unethical behavior as a leader: Literature shows that when directors, managers, and other important people in an organization do not show the correct behavior, it is also easy for other employees to use this as an excuse to behave with less integrity and to make less ethical considerations. (CO019)

Five compliance officers emphasized that it is important that managers are an example in being open: they have to be open about their own mistakes, doubts, and dilemmas. This will set the example for other employees to do the same. Consequentially, they will discuss their dilemmas and mistakes more easily and learn from them. This will prevent future misconduct and contribute to a safe work environment.

### Discussing Rules and Behavior

Seventeen participants mentioned that they think they can change behavior by discussing rules and behavior with employees. According to the compliance officers, having a conversation or discussion can change behavior in different ways. First, it provides the opportunity to discuss gray areas and dilemmas that occur in the workplace: it allows for the discussion of different perspectives. CO005 explained: If you have a question in that area, or the question: ‘How do we interact with each other?’ Or about what you can do with a client—that often involves gray area issues. To have open conversations about that with coworkers. Or to discuss it with the compliance department, if you want to. But it is not weird to have questions about these things, and sometimes things can be a bit gray—the whole concept: dilemmas.

Additionally, discussion allows compliance officers and managers to explain rules or expected behavior to employees. This can increase the knowledge of rules and help with the understanding of rules within the organization. According to CO015, discussing the rules may trigger someone’s intrinsic behavior to do the right thing: You hope to trigger that intrinsic motivation with that conversation, the personal approach, the explanation of why, but not to only focus on the literal rule. You hope to achieve that in that way. Sometimes it is possible, and sometimes it is impossible, I think, but with some subjects or some behaviors it is an option.

Furthermore, (possible) misconduct can also be discussed. Such, often more formal, discussions and conversations can help to provide more insights into someone’s behavior. On the one hand, the employee can gain insight into their own behavior, for example when they show inappropriate conduct, they are not aware of. On the other hand, it allows the compliance officer to gain a better understanding of the motivations behind certain behavior. This can be relevant when deciding whether someone needs to be sanctioned or deserves a second chance. Four compliance officers described how such conversations can also have a more deterrent effect. CO014 said: “Often a good conversation with the manager is already enough, and often the employee feels horrible about the mistake.”

Although compliance officers see discussion as a good mechanism to prevent misconduct, it is not always possible for them to have such a discussion. CO015 explained: “What works best is to engage in conversation, but people generally not have so much time for this.” Thirteen compliance officers mentioned that more informal discussions should also happen in settings other than solely between an employee and a manager or CO. Employees should also start conversations among themselves. Ideally, employees should also start conversations with compliance officers when they need advice, and even with managers. This would help to create an open culture, which stimulates more compliant behavior. The idea is that by discussing situations on a regular basis and in different settings, people will feel safe enough to ask for advice and learn from their mistakes, which will ultimately lead to an organization that wants to learn, and to less misconduct.

Discussing rules and behavior is closely related to reporting. However, compliance officers see discussions as more accessible, as discussion is not a formal instrument. However, discussing rules and behavior can lead to formal reporting, and reports can lead to discussing rules and behavior.

### Understanding Rules

Eleven compliance officers think that understanding rules can lead to less organizational misconduct. There is much overlap between understanding rules and knowledge of rules: only one compliance officer did not explicitly mention knowledge of rules as a mechanism. The compliance officers emphasized that, for them, understanding rules is a crucial step toward better compliance: if there is better understanding of the rules, there will be more compliance. During the interviews, three different meanings of understanding rules were mentioned. For five compliance officers, understanding rules meant that their aim is to translate legal texts and laws into more understandable texts for employees who are not legally trained, and to explain what this entails. This will make it easier for employees to understand what it is they have to comply with. Three compliance officers emphasized that it is important that employees understand the “why” behind a rule or policy. As CO015 explained: And I think that it is an important part of the training you give. If someone starts working at a company, you should not only explain: ‘These are the rules,’ but particularly why we have these rules and why it is important to behave this way. Just an explanation of the rule itself does not do much.

Last, five compliance officers explained that, for them, it is important that people understand the required behavior: only if employees know what that behavior entails can they show it. CO007 stated: What does that mean? What do we mean by that? What do we want to see? So, you can only steer behavior if you know what that behavior should be. That is the first step. If that is not clear, you can never steer it.

Thus, for these compliance officers, understanding rules is about understanding the meaning of the rule, the behavior that it requires, and why it exists. They saw different possibilities for enhancing the understanding of rules, namely through e-learning or trainings, during conversations and discussions with employees and teams, or during official events such as swearing-in ceremonies.

### System Architecture

Nine compliance officers mentioned that the architecture of internal systems and processes can influence organizational misconduct. CO021 explained: Then you will have to implement a change in system or behavior: compliant behavior can also require a systemic change. It is not always behavior. Sometimes, we just need to add a checkbox to the system, or put an extra module in the system, which prevents you from continuing. With that, you can also stimulate compliant behavior.

Eight compliance officers explained that with internal processes, they can make it easier for employees to do the right thing and more difficult to commit misconduct. An example that was used multiple times is the four-eyes principle to minimize the possibility of fraud, where an activity such as a transaction needs to be approved by two people. With these systems and processes, the compliance officer can simply “facilitate” (CO021) the employee to comply. The participants were divided on whether employees should understand why the processes exist and therefore understand the law they have to comply with. On the one hand, participants explained how such processes and systems are especially well suited for compliance with strict laws and policies: the employee does not even have to understand the exact law they are complying with. Conversely, CO001 emphasized that having the internal processes is not enough; employees also need to understand why the process exists.

Contrary to the possible positive effects on organizational misconduct, one compliance officer described how they think that internal processes and systems can actually have negative consequences: they can lead to frustration, and complex systems with many boxes to check can easily lead to (unintentional) mistakes. CO014 explained: And for the employee, if they have to deal with the systems, there is a lot of frustration. Look, if those 38 screens have to be opened to answer one question, then you know for sure that it will go wrong somewhere, in copying, in dragging. So, you should not be surprised, then, that a mistake will occur.

### Reporting

In total, eight compliance officers mentioned that having a reporting system or whistleblowing system is a mechanism through which they think they can change behavior. According to the compliance officers, it is important to know what is happening within the organization in order to be able to mitigate misconduct. As CO012 said: “I think that prevention is most important, but if it goes wrong, you have to be well organized.” The compliance officers believe that reports allow them to detect possible misconduct and stop it before it grows within the organization. One compliance officer explained: Look, when the reports stay concealed, you do not have any idea what is going on in the organization regarding inappropriate conduct and integrity. And then, you risk such a culture growing in areas of the organization, or even in the whole organization. (CO012)

The idea is that, based on a report, a proper investigation can be done, looking into what went wrong and how this could have happened. CO017 explained: “You want to take reports seriously, take them, investigate them, and give feedback, then draw a conclusion and learn from them.” Depending on the results of the investigation, compliance officers can rely on other mechanisms, such as sanctioning or changing internal systems, to change the unwanted behavior. This depends on the type of misconduct but also on the motivation behind the misconduct.

Seven compliance officers described that how the report is handled is important for behavioral change. On the one hand, they have to protect the reporter/whistleblower because the risk is that no one will report anything if they doubt their safety, which will lead to fewer insights into what is happening in the organization. On the other hand, they have to be transparent enough with others to prevent gossip or an unsafe culture. CO005 explained that the ultimate goal is to create “a speak-up culture,” where everyone would report misconduct.

## Footnotes

1 In several nonprofit or governmental organizations, compliance (management) is referred to as integrity (management). In this paper, the term compliance refers to both compliance and integrity.

2 By ‘experience’, I refer not merely to the number of years an individual has spent as a compliance officer, but to the broader, more subjective sense of engagement with and familiarity in performing the work.

3 This research was conducted as part of an ERC project about the behavioral assumptions of legal practitioners. It encompassed multiple qualitative research projects that were conducted in parallel. The researchers responsible for each of these subprojects collaborated in the development of the core interview materials, which were then further adjusted to each project’s unique focus.

4 There were other mechanisms mentioned by compliance officers, but only sporadically. To give a clear representation of the sample, only mechanisms that are mentioned more than five times will be discussed in this paper.

5 It should be noted that this paper does not provide a complete overview of all the literature on role modeling and leadership in the organizational setting as this is beyond the scope of this paper.
